# Preparation and Properties of Polyimide Composite Membrane with High Transmittance and Surface Hydrophobicity for Lightweight Optical System

**DOI:** 10.3390/membranes12060592

**Published:** 2022-06-03

**Authors:** Jiajia Yin, Haohao Hui, Bin Fan, Jiang Bian, Junfeng Du, Hu Yang

**Affiliations:** 1 Institute of Optics and Electronics, Chinese Academy of Sciences, Chengdu 610209, China; dream2001hui@163.com (B.F.); bianjiang007@126.com (J.B.); bethleham@163.com (J.D.); valleytramp@163.com (H.Y.); 2 Laser Fusion Research Center, China Academy of Engineering Physics, Chengdu 610200, China; dream2001hiu@163.com

**Keywords:** polyimide composite membrane, high transmittance, hydrophobic surface

## Abstract

Polyimide membranes have excellent physiochemical properties which make them valuable materials for optical area. However, common aromatic polyimide membrane trend to show low transmittance in visible region because of the charge-transfer complex (CTC) in molecular structures. Moreover, it’s trending to show high moisture uptakes because of the hydrophilic imide rings in molecular structure. In this work, a polyimide composite membrane with SiO_2_ antireflective membrane on both sides was prepared. High transmittance (93% within 500~800 nm) and surface hydrophobicity was realized simultaneously. The polyimide composite membrane showed great optical homogeneity. The SiO_2_ antireflective membranes on polyimide substrate were prepared through a simple and efficient sol-gel method. The surface roughness of polyimide membrane substrate on each side has been improved to 1.56 nm and 3.14 nm, respectively. Moreover, the excellent thermal stability and mechanical property of polyimide membrane has been preserved, which greatly improves the range of applications for the composite membrane. It is a good candidate for light weight optical system.

## 1. Introduction

Polyimides (PIs) are aromatic heterocyclic polymer. They have been widely used in aviation, aerospace, nuclear power, and microelectronics because of their superior thermal, mechanical, and chemical resistance properties [1,2]. They are also attractive lightweight mirror substrate material due to their low surface density. However, common aromatic polyimide materials trend to show low transmittance in visible region because of the charge-transfer complex (CTC) in molecular structures [3,4,5]. Moreover, it trending to show high moisture uptakes because of the hydrophilic imide rings in molecular structure [6,7,8]. These problems limit its applications in optics. High performance PI membrane with good transmittance, optical homogeneity, and surface hydrophobicity is urgently needed for lightweight optical system. At present, many membranes optical systems are being explored. In inertial confinement fusion physics experiments, ultrathin polymer membranes are commonly used targets for the National Ignition Facility (NIF), typical as various windows or as tents [9]. In MOIRE (Membrane Optical Imager Real-time Exploitation) program, polyimide membrane has been used as membrane optic for space telescopes. In Falcon SAT-7 system, polyimide membrane has been developed as a solar telescope [10,11].

Our previous work mainly focused on the modification of polyimide molecular structure to reach the goal [12]. But in many research, multilayer polymer-inorganic composites have been studied extensively to improve the performance of polymer substrate. Inorganic layer on the polymer membrane can act like a complementary coating component. It will provide desirable properties such as high transmittance, electrical conductivity, and thermal conductivity to polymers [13,14]. SiO_2_ membrane has been commonly used in mirror-based materials as antireflective material and help to form a hydrophobic surface [15,16,17]. Fabrication of antireflective SiO_2_ membrane on inorganic substrate by sol-gel method has been widely used. Compared with traditional physical vapor deposition [18], chemical vapor deposition [19], sol-gel method is cheaper and more convenient for large area coating. The antireflective membrane fabricated by sol-gel method also showed good optical performance. It has been used in fabrication of high laser damage threshold SiO_2_ antireflective membrane for a long time [20,21].

Herein, in this work we focused on the fabrication of SiO_2_-polyimide-SiO_2_ composite membrane by simple and efficient sol-gel method. The SiO_2_ layer on both sides of polyimide membrane with good uniformity has been successfully coated. The optical, hydrophilic/hydrophobic, thermal and mechanical properties have been compared to help understand the effects of the SiO_2_ layer.

## 2. Materials and Methods

### 2.1. Materials

4,4′-Diaminobenzanilide (DABA, 98%), 4,4′-diamino-2,2′-Dimethylbiphenyl (TMDB, 98%), 3,3′,4,4′-biphenyltetracarboxylic dianhydride (BPDA, 98%) and N,N-dimethylacetamide (DMAC, 99%) were purchased from TCI reagents (Shanghai, China). 3-(Trimethoxysilyl)propyl methacrylate (MPS, 97%), hydrochloride acid (HCl), tetraethyl orthosilicate (TEOS, 98%), ethanol (99.8%), n-butanol(99.8%), ammonium hydroxide solution (28%), 2-hydroxy-2-methylpropiophenone (97%) were purchased from Aladin reagents (Shanghai, China). BPDA was dried at 180 °C in a vacuum (Reale, Dongguan, China) for 24 h prior to use. DMAC was purified by distillation under reduced pressure and dehydrated with 4 Å molecular sieves prior to use. Other solvents and regents were used as received.

### 2.2. Preparation of Polyimide Membrane

**Figure 1:**
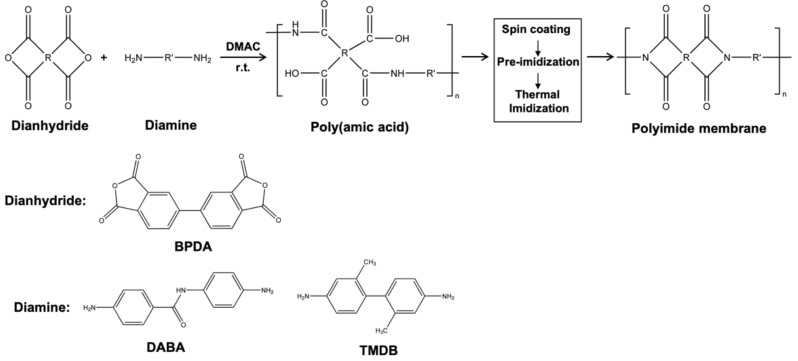
The synthesis of Poly(amic acid) and preparation process of PI membrane.

Poly(amic acid) resin was synthesized by the polyaddition of equimolar amounts of diamine (DABA/TMDB; molar ratio DABA:TMDB = 1:1, n_DABA_ = n_TMDB_ =0.05 mol) and dianhydride (BPDA, n_DABA_ = 0.10 mol). Then, PI membrane was prepared followed by thermal imidization. In the experiment, PI membrane was prepared according to the following procedure. Diamine DABA/TMDB were dissolved in DMAc (m_DMAC_ = 500 g) in a dry three neck flask equipped with a mechanical stirrer (Taihong-sheng, Zhengzhou, China) and nitrogen flow under room temperature. Then, dianhydride BPDA was added to the solution in batches with continuous stirring. The reaction mixture was stirred for 30 min at 0 °C and then left to react overnight at room temperature. The concentration of the solution is controlled around 8~10% (wt.). The homogeneous and viscous PAA resin was produced with a high inherent viscosity of 1.34–1.75 dL/g, which indicates that the polymeric precursor has a relatively high molecular weight. The PAA resin was filtrated and deaerated before casting on the surface of quartz glass plate by spin coating (Leibo, Jiangsu, China). The wet membrane is pre-imidized by a hot plate under 70 °C for 30 min to remove excess solvent. Then, it was heated by a vacuum oven (Reale, Dongguan, China) in stages (100 °C/1 h; 200 °C/1 h; 350 °C/1 h) to elevated temperatures to further remove solvent and convert the amic acid functional groups to imides with a cyclodehydration reaction. The temperature rise and fall processes are optimized based on the previous study [12,22] to ensure the full imidization. The thickness of the membrane is around 20 µm. The produced polyimide membrane was annealed at an established temperature in a vacuum oven to release the residual stress. The synthesis of poly(amic acid) and preparation process of PI membrane are shown in Figure 1.

### 2.3. Preparation of Polyimide/SiO_2_ Composite Membrane

#### 2.3.1. Preparation of UV-Curable Silicone Pre-Polymer Sol

Using MPS [3-(trimethoxysilyl) propyl methacrylate] as precursor and HCl (0.01 mol/L) as catalyst for preparing pre-polymer sol. The molar ratio of MPS to HCl is 1:3. After the hydrolysis and polycondensation reaction of MPS, the UV-curable silicone pre-polymer sol was prepared.

#### 2.3.2. Preparation of Colloidal Silica Sol

The colloidal silica sol was prepared by Stöber method. Mix TEOS, absolute ethanol and 28% NH_3_·H_2_O with the mass ratio of 1:32:0.25 under 5 °C for 5 h. The solution was kept still in a sealed glass container for 8~10 days at room temperature for aging process. Then it was refluxed for 10 h to remove the extra ammonia. The concentrate of the colloidal suspension is 3% (wt.).

#### 2.3.3. Preparation of UV-Curable Silica Sol

Mix UV-curable silicone pre-polymer sol (1 g), colloidal silica sol (60 g), n-butyl alcohol (25 g), ethanol (22 g), and 2-hydroxy-2-methylpropiophenone (1 g) by magnetic stirring for 3 h to get UV-curable silica sol. The concentration of silica is about 4% (wt.). Filter it through a 0.2 μm polyvinylidene fluoride (PVDF) membrane for final use.

#### 2.3.4. Preparation of SiO_2_ Antireflective Membrane on Polyimide

**Figure 2:**
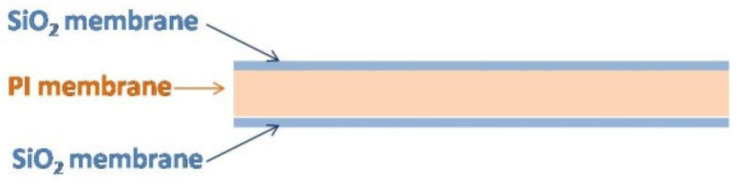
The schematic structure diagram of multilayer composite membrane.

Before coating PI membrane with UV-Curable silica sol, clean both sides of it by ethanol and toluene. It was coated with SiO_2_ membrane by dip-pulling method with the pulling speed of 1.6 mm/s. Then, the coated polyimide membrane experienced a 60 s UV irradiation from an Hg lamp on both sides. The UV light intensity was measured (EIT UVICURE Plus II, EIT, UAS) to be 200 mW/cm^2^. The thickness of SiO_2_ membrane on each side of PI membrane is around 80 nm. The schematic structure of polyimide composite membrane is shown in Figure 2. For optical element with antireflective membrane, when the refractive index meets the requirements of $n_{1}=\sqrt{n_{0}n_{2}}$ (*n*_1_ is the refractive index of antireflective membrane, *n*_0_ = 1 is the refractive index of air, *n*_2_ is the refractive index of substrate) [23], then the optical element shows the highest transmittance. In this research, the refractive index of PI membrane is 1.63. According to the requirement, the refractive index of SiO_2_ antireflective membrane is optimized to 1.28 to meet the requirements.

### 2.4. Characterization

The thickness of the polyimide membrane was measured by using a commercially available spectral reflectometer (Membraneetrics-F20 thin film analyzer, Membraneetrics Inc., San Diego, CA, USA). The transmission spectra of membranes (22 µm thickness) were measured by using ultraviolet-visible-near infrared spectrophotometer (Lambda 1050, Perkin Elmer, Waltham, MA, USA) in the wavelength (λ) range of 200~2500 nm. The optical inhomogeneity of membranes was characterized by using a Zygo laser interferometer (GPI XP, Middlefield, CT, USA) with an accuracy of λ/1000 (λ = 632.8 nm, λ is the measurement wavelength). The surface roughness was measured by laser interferometer optical microscope (Bruker Optics, Ettlingen, Germany). The contact angle tests were performed with a Dataphysics OCA 25 optical contact angle system (Dataphysics, Germany) and determined as Chinese national standard of GB/T 30,693–2014 with dimension of 3 mm × 30 mm. The thermal stability of PI membranes was evaluated by thermo gravimetric analysis (TGA), which were performed on a TG 209 F1 Libra (Netzsch, Selb, Germany) at a heating rate of 20 K/min in a nitrogen atmosphere. The dynamic mechanical analysis (DMA) was performed on a TA Q800 instrument (TA Instruments, New Castle, DE, USA). The glass transition temperature (T_g_) of PI specimens was regarded as the peak temperature of the tan δ curves. The test parameters are as follows: heating rate is 5 °C/min, load frequency is 1 Hz, the atmosphere is nitrogen. The tensile properties of PI membranes were measured on an Instron-5944 tensile apparatus (Norwood, MA, USA). The samples size is 150 (l) mm × 20 (w) mm × 0.022 (h) mm. Measurement was taken at room temperature in accordance with the Chinese national standard of GB/T1040.3-2006 at a drawing rate of 10 mm/min. FTIR spectra were measured on a Fourier transform spectrophotometer (Bruker, Germany) by KBr pellet method for bare PI membrane and composite PI membrane.

## 3. Results and Discussion

### 3.1. Optical Properties

**Figure 3:**
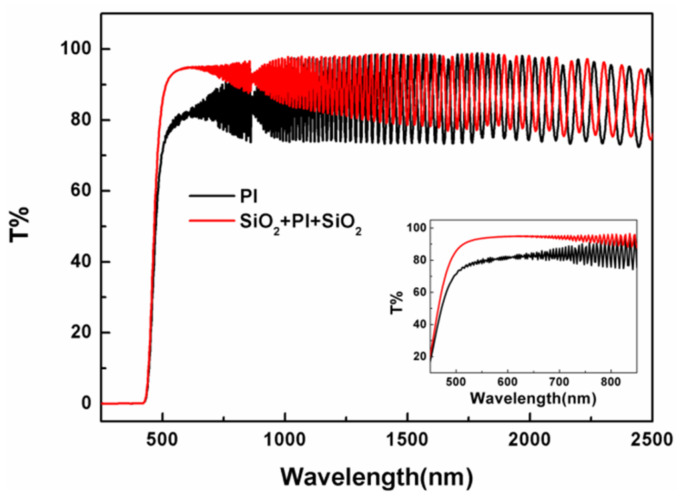
The transmittance spectra of PI membrane before and after coated with SiO_2_ antireflective membrane on both sides. Insert picture is the enlarge part of the spectra at wavelength between 450 nm and 900 nm.

**Figure 4:**
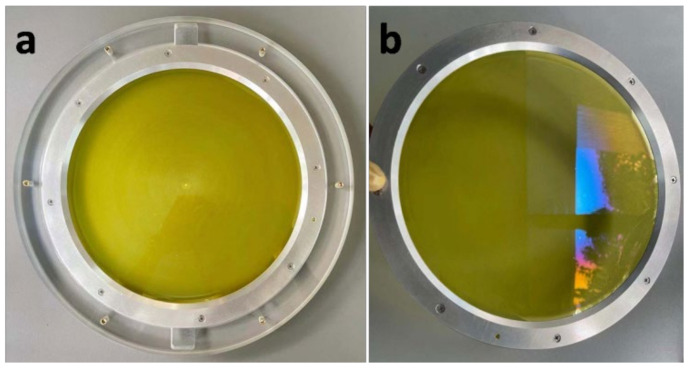
The pictures of Φ200 mm PI membrane under sunlight (**a**) before and (**b**) after coated with SiO_2_ antireflective membrane on both sides.

The optical effects of SiO_2_ antireflective membranes on PI membrane were investigated by the optical transmittance and wavefront error measurements. The ultraviolet-visible (UV–Vis) spectra of the PI membrane (thickness = 20 µm) before and after coating with SiO_2_ antireflective membrane were measured and shown in Figure 3. The results were listed in Table 1. The transmittance of the PI composite membrane improved significantly with the peak of 95%, increased by 13% than the bare PI membrane at the wavelength of 606 nm. In the visible region of 500~800 nm, the average transmittance of composite membrane is 93%, while for the bare PI membrane it is only 81%. The wavelength at 50% transmittance for bare PI membrane is 472 nm. While the wavelength at 50% transmittance for composite PI membrane blue shifted to 466 nm. As shown in Figure 4, after coating with SiO_2_ antireflective membrane, the Φ200 mm PI membrane shows a bluepurple reflection under sunlight.

**Figure 5:**
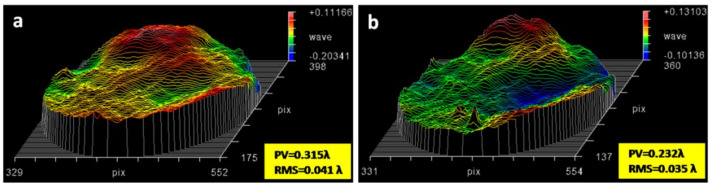
The wavefront error of Φ200 mm PI membrane (**a**) before and (**b**) after coated with SiO_2_ antireflective membrane on both sides.

Optical homogeneity is also a key factor for optical lens. In order to develop high performance PI membranes as lightweight optical lens, the PI membrane must possess optical homogeneity at the same order of magnitude to inorganic glass substrates. Principally, optical thickness aberration would have a negative modulation effect on the ideal light wavefront, so optical inhomogeneity of the membrane lens can be characterized by wavefront errors with the application of the Zygo laser interferometer. The thickness aberration of bare PI and composite PI membranes with 20 μm thickness and Φ200 mm aperture has been tested. As shown in Figure 5, the composite PI membrane achieved good thickness uniformity with wave-front error around λ/30 in RMS (root mean square), which improved by 14.6% than bare PI membrane.

### 3.2. Surface Roughness and Water Absorption

**Figure 6:**
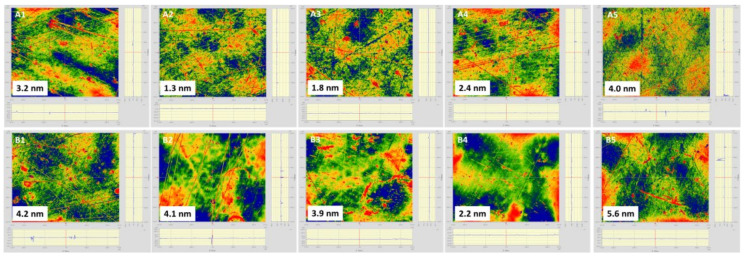
The surface roughness of bare PI membrane on side A and side B.

**Figure 7:**
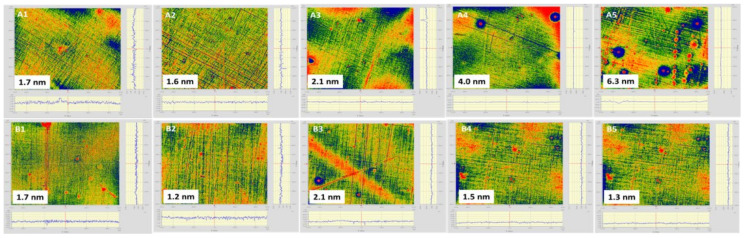
The surface roughness (Sq, nm) of PI membrane on side A and side B after coated with SiO_2_ antireflective membrane on both sides.

High-quality optical systems need minimized scattering optical module to realize high power output and low loss. Surface roughness is a key factor for optical lens in optical system. A membrane lens with low surface roughness will promote light weight, high integrated optical system development. As shown in Figure 6, bare PI membrane owns an average Sq (root mean square height standard deviation of the height distribution on surface) of 2.54 nm on side A and an average Sq of 4 nm on side B. After coating with SiO_2_ antireflective membrane on both sides, composite PI membrane showed a surface with lower roughness. As shown in Figure 7, the average surface roughness (Sq) on side A decreased by 39%, which is 1.56 nm. The average surface roughness (Sq) on side B instead decreased by 22%, which is 3.14 nm. Detailed information is in Table 2. The SiO_2_ antireflective membrane not only helps to decrease the surface light scattering and also improves the transmittance of the PI membrane.

**Figure 8:**
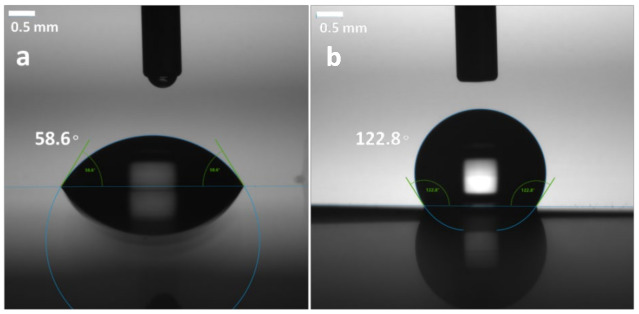
Water contact angle of PI membrane before and after coated with SiO_2_ antireflective membrane.

The sessile drop method was employed to evaluate the membrane surface wettability, where deionized water was used as the testing liquid. In general, polyimide mostly had higher water absorption than common polymers due to the hydrophilic nature of imide rings and some hydrophilic moieties (ether, sulfone and carbonyl group, etc.) in the molecular backbones [6]. So, a simple and efficient way to decrease the water absorption of PI membrane was urgently required. The contact angle between the dropped water and the membranes surface was measured as shown in Figure 8, after coating with SiO_2_ antireflective membrane, the surface energy of PI composite membrane decreased greatly. As a result, the static water contact angle is improved from 58.6° to 122.8°. The sessile drop method was also employed on K9 glass and silicon the same SiO_2_ antireflective membrane (the results have been shown in Supplementary Materials Figures S1 and S2). On the K9 the static water contact angle is improved from 59.9 to 130.1°. On the silicon substrate, the static water contact angle is improved from 66.5 to 131.8°. In general, nanoporous SiO_2_ thin membranes made by sol-gel process are an important platform for controlling the wettability of optic lens surface [24,25]. In this research, SiO_2_ antireflective membrane not only can help to improve the optical properties, surface roughness of PI substrate but also can be used as water vapor isolation layer.

### 3.3. Thermal and Mechanical Properties

**Figure 9:**
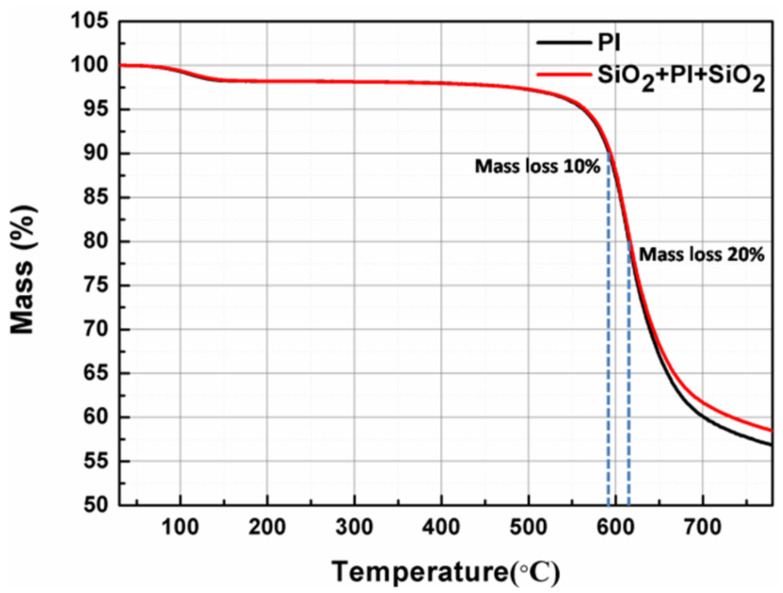
The TGA curves of PI membranes before and after coated with SiO_2_ antireflective membrane in nitrogen.

**Figure 10:**
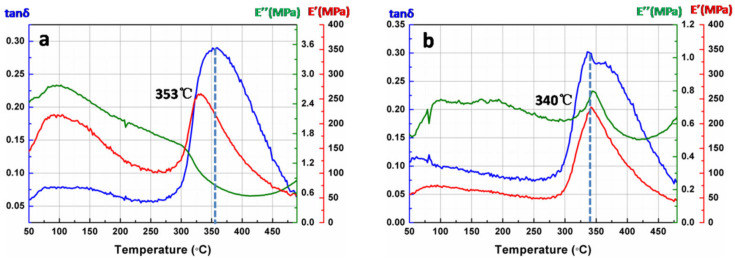
The DMA results of PI membranes (**a**) before and (**b**) after coated with SiO_2_ antireflective membrane in nitrogen.

Thermo gravimetric analysis is used to evaluate the thermal stability of membranes over high temperatures. Figure 9 shows the TGA curves of the PI membranes before and after coating with SiO_2_ antireflective membrane. Clearly, the decomposition temperature of the bare PI membrane and PI composite membrane are all up to 500 °C, the temperature at 10%/20% mass loss of the two samples are almost the same. While under the protection of SiO_2_ antireflective membrane, the PI composite membrane shows a higher residual weight (59.4%) retention than bare PI membrane at 750 °C. Dynamic mechanical analysis is used to determine the T_g_ of bare PI membrane and PI composite membrane. Figure 10 shows the DMA curves of the PI membranes before and after coating with SiO_2_ antireflective membrane. All the two samples exhibited high heat-resistant properties with T_g_ values of 353 °C and 340 °C, respectively. The difference between the bare PI and the PI composite can be attributed to the SiO_2_ antireflective membrane on both surfaces of PI. According to the results, after coating with antireflective membrane the excellent thermal properties of the bare PI membrane has been preserved. The results were summarized in Table 3.

**Figure 11:**
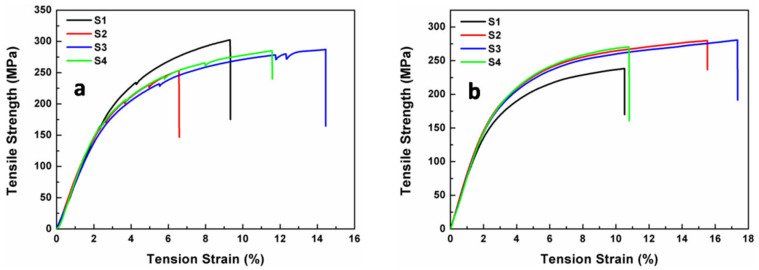
Mechanical properties of PI membranes (**a**) before and (**b**) after coated with SiO_2_ antireflective membrane. Each sample has been tested for four times to guarantee the accuracy.

The mechanical properties of the PI membranes including tensile strengths (T_S_), Young’s modulus (T_M_), and elongations at breakage (ɛ_b_) were systematically investigated and summarized in Table 4. All the PI membranes exhibited excellent mechanical properties with TS values above 250 MPa and ɛ_b_ values above 10%. As shown in Figure 11, bare PI membrane showed a slightly higher tensile strength (282 MPa) than the one after coating with SiO_2_ antireflective membrane (267 MPa). While, PI composite membrane showed a higher average elongation at break (13.5%) than bare PI membrane (10.5%). The minor differences can be caused by the SiO_2_ sol-gel coating process. The UV-curable silica sol solution contains a certain amount of ammonium hydroxide solution. During the dip-pulling process, PI membrane needs to be soaking into the UV-Curable silica sol for a few seconds. The hydroxyl influenced the surface structure of PI membrane. But the original strong tensile properties are retained.

### 3.4. Chemical Structures

**Figure 12:**
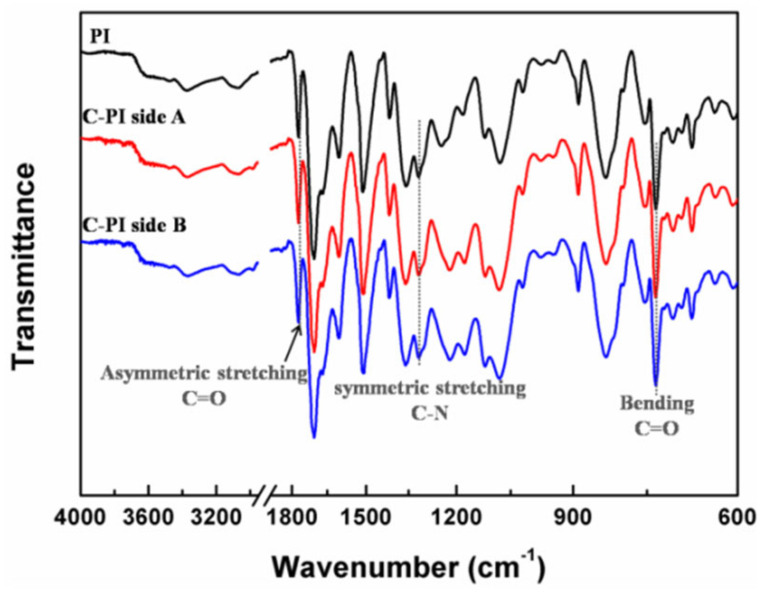
FTIR spectra of PI membranes before and after coated with SiO_2_ antireflective membrane. The FTIR spectras of PI composite membrane have been tested on both sides.

The chemical structures of PI membrane and PI composite membrane were characterized by FTIR spectra, as shown in Figure 12. The PI composite membrane has been tested on both sides. There is no 1650 cm^−1^ (C–NH) characteristic absorption band found in the three spectra, which indicates the full imidization of PAA and condensation. The characteristics of the PI are further evidenced by the following absorption bands 1775 cm^−1^ (C=O asymmetric stretching), 1320 cm^−1^ (C–N symmetric stretching), and 730 cm^−1^ (C=O bending). There is no obvious absorption band of Si–O–Si, which can be affected by the strong vibration peak of polyimide membrane.

## 4. Conclusions

The novel polyimide composite membrane which is composed of polyimide membrane substrate and antireflective SiO_2_ was successfully prepared via a simple and efficient sol-gel method. High transmittance (93% within 500~800 nm) and surface hydrophobicity (static water contact angle: 122.8°) were realized simultaneously. The polyimide composite membrane showed good thickness uniformity with wave-front error around λ/30 in RMS (root mean square), which improved by 14.6% compared to polyimide membrane substrate. The surface roughness of polyimide membrane substrate also improved. In addition, the excellent thermal stability and mechanical property of polyimide membrane substrate were preserved, which greatly improved the range of applications for the composite membrane. The novel polyimide composite membrane is a good candidate for high performance lightweight optical systems.

## Figures and Tables

**Table 1:** The transmittance results of PI membrane before and after coated with SiO_2_ antireflective membrane on both sides.

Sample	T_606 nm_^a^	λ_T50%_^b^	T_AV(500~800 nm)_^c^
SiO_2_ + PI + SiO_2_	95%	466 nm	93%
PI	82%	472 nm	81%

^a^ T_606_: the transmittance at 606 nm; ^b^ λ_T50%_: the wavelength at 50% transmittance; ^c^ T_AV (500~800 nm)_: average transmittance between 500 to 800 nm.

**Table 2:** The surface roughness results (Sq, nm) of PI membrane on side A and side B before and after coated with SiO_2_antireflective membrane on both sides.

Sample	Sq ^a^ (Side A, nm)	Average	σ_A_	Sq (Side B, nm)	σ_B_	Average
PI	3.2/1.3/1.8/2.4/4.0	2.54 nm	0.96 nm	4.2/4.1/3.9/2.2/5.6	0.32 nm	4.00 nm
SiO_2_ + PI + SiO_2_	1.7/1.2/2.1/1.5/1.3	1.56 nm	1.08 nm	1.7/1.6/2.1/4.0/6.3	1.80 nm	3.14 nm

^a^ Sq: surface roughness (root mean square); σ: standard deviation.

**Table 3:** Thermal properties of membranes before and after coated with SiO_2_ antireflective membrane.

Sample	T_10%_^a^	T_20%_^b^	Carbon Yield_(750 °C)_	T_g_^c^
PI	591 °C	614 °C	57.7%	353 °C
SiO_2_ + PI + SiO_2_	593 °C	616 °C	59.4%	340 °C

^a^ T_10%_: the temperature at 10% mass loss; ^b^ T_20%_: the temperature at 20% mass loss; ^c^ T_g_: the glass transition temperature.

**Table 4:** Mechanical properties of PI membranes before and after coated with SiO_2_ antireflective membrane.

Sample	T_S_^a^ (MPa)	T_M_^b^ (GPa)	Ɛ_b_^c^ (%)
PI	282	8	10.5
SiO_2_ + PI + SiO_2_	267	8	13.5

^a^ T_S_ average tensile strength; ^b^ T_M_: average tensile modulus; ^c^ ɛ: average elongation at break.

## Data Availability

The data presented in this study are available on request from the corresponding author.

## Supplementary Materials

The following supporting information can be downloaded at: https://www.mdpi.com/article/10.3390/membranes12060592/s1, Figure S1: Water contact angle of K9 glass before and after coated with SiO_2_ antireflective film.; Figure S2: Water contact angle of Silicon substrate before and after coated with SiO_2_ antireflective film.

## References

[1] Li T.L., Hsu S.L.C. (2010). Enhanced thermal conductivity of polyimide membranes via a hybrid of micro-and nano-sized boron nitride. J. Phys. Chem. B.

[2] Gao H., Yorifuji D., Wakita J., Jiang Z.H., Ando S. (2010). In situ preparation of nano ZnO/hyperbranched polyimide hybrid membrane and their optical properties. Polymer.

[3] Ando S., Matsuura T., Sasaki S. (1997). Coloration of aromatic polyimides and electronic properties of their source materials. Polym. J..

[4] Hasegawa M., Horie K. (2001). Photophysics, photochemistry, and optical properties of polyimides. Prog. Polym. Sci..

[5] Ke F., Song N., Liang D., Xu H. (2013). A method to break charge transfer complex of polyimide: A study on solution behavior. J. Appl. Polym. Sci..

[6] Wu T., Dong J., Gan F., Fang Y., Zhao X., Zhang Q. (2018). Low dielectric constant and moisture-resistant polyimide aerogels containing trifluoromethyl pendent groups. Appl. Surf. Sci..

[7] Chen W., Liu F., Ji M., Yang S. (2016). Synthesis and Characterization of Low-CTE Polyimide Films Containing Trifluoromethyl Groups with Water-repellant Characteristics. High Perform. Polym..

[8] Fujita S., Kamei Y. Electrical properties of polyimide with water absorption. Proceedings of the 11th International Symposium on Electrets.

[9] Didier M., Bruno D., Benoit B., Lavergne Y., Thevenot G. (2000). High performance aromatic polyimides for inertial confinement fusion experiments. Polym. Int..

[10] Rahlves M., Rezem M., Boroz K., Schlangen S., Reithmeier E., Roth B. (2015). Flexible, fast, and low-cost production process for polymer based diffractive optics. Opt. Express.

[11] Tracy L., Copp J.L.D., Paul A., Bill T., Jeff K. MOIRE: Membrane Material Property Characterizations, Testing and Lessons Learned. Proceedings of the Spacecraft Structures Conference.

[12] Yin J., Mao D., Fan B. (2021). Copolyamide-Imide Membrane with Low CTE and CME for Potential Space Optical Applications. Polymers.

[13] Lü C., Yang B. (2009). High refractive index organic–inorganic nanocomposites: Design, synthesis and application. J. Mater. Chem..

[14] Jones W.E., Chiguma J., Johnson E., Pachamuthu A., Santos D. (2010). Electrically and thermally conducting nanocomposites for electronic applications. Materials.

[15] Dou W., Wang P., Zhang D., Yu J. (2015). An efficient way to prepare hydrophobic antireflective SiO_2_ membrane by Sol-gel Method. Mater. Lett..

[16] Purcar V., Stamatin I., Cinteza O., Petcu C., Raditoiu V., Ghiurea M., Miclaus T., Andronie A. (2012). Fabrication of hydrophobic and antireflective coatings based on hybrid silica membranes by solgel process. Surf. Coat. Technol..

[17] Li H., Li N., Zhang Y., He H., Liu Z. (2017). Anti-reflection OTS-treated SiO_2_ thin membranes with super- hydrophobic property. J. Sol-Gel Sci. Technol..

[18] Picas J.A., Forn A., Baile M.T., Martín E. (2005). Substrate effect on the mechanical and tribological properties of arc plasma physical vapour deposition coatings. Int. J. Refract. Met. Hard Mater..

[19] Porporati A., Roitti S., Sbaizero O. (2003). Metallorganic chemical vapor deposition of Ta_2_O_5_ membranes. J. Eur. Ceram. Soc..

[20] Roux R. (1995). High-energy lasers sol-gel coating benefits megajoule laser. Laser Focus World.

[21] Floch H.G., Priotton J.J. (1990). Colloidal sol-gel coating. Am. Ceram. Soc. Bull..

[22] Chen W., Chen W., Zhang B., Yang S., Liu C.Y. (2017). Thermal imidization process of polyimide membrane: Interplay between solvent evaporation and imidization. Polymer.

[23] Macleod H.A., Pike E.R., Brown R.G.W. (2010). Antireflection Coating. Thin-Film Optical Filters.

[24] Jeong H.J., Kim D.K., Lee S.B., Kwon S.H., Kadono K. (2001). Preparation of water-repellent glass by sol–gel process using perfluoroalkylsilane and tetraethoxysilane. J. Colloid Interface Sci..

[25] Bibo X., Jianhui L., Yuanyang L., Bowen Y., Shuming Z., Bo J. (2017). Preparation of sponge-like porous SiO_2_ antireflective coatings with excellent environment-resistance by an acid-catalysed sol–gel method. RSC Adv..

