# Supplementary material for: Preparation and Properties of Polyimide Composite Membrane with High Transmittance and Surface Hydrophobicity for Lightweight Optical System
Source: Membranes (Basel). 2022 Jun 3;12(6):592. doi: 10.3390/membranes12060592 (PMC9230139; doi:10.3390/membranes12060592)
Supplement: Supplementary file 1 [file membranes-12-00592-s001.zip › membranes-1736822-supplementary.pdf]

Supplementary Materials

Preparation and Properties of Polyimide Composite Membrane with High Transmittance and Surface Hydrophobicity for Lightweight Optical System

Jiajia Yin [1,*,†], Haohao Hui [2,†], Bin Fan [1], Jiang Bian [1], Junfeng Du [1] and Hu Yang [1]

[1] Institute of Optics and Electronics, Chinese Academy of Sciences, Chengdu 610209, China; dream2001hui@163.com (B.F.); bianjiang007@126.com (J.B.); bethleham@163.com (J.D.); valleytramp@163.com (H.Y.)

[2] Laser Fusion Research Center, China Academy of Engineering Physics, Chengdu 610200, China; dream2001hiu@163.com

* Correspondence: yinjj@ioe.ac.cn; Tel.: +86-028-6413-6346

† Author Jiajia Yin and Haohao Hui contributed equally to this work.

The preparation technics of the SiO_2 antireflective film on K9 glass and silicon are the same. On the K9 the static water contact angle is improved from 59.9 to 130.1°. On the silicon substrate, the static water contact angle is improved from 66.5 to 131.8°.

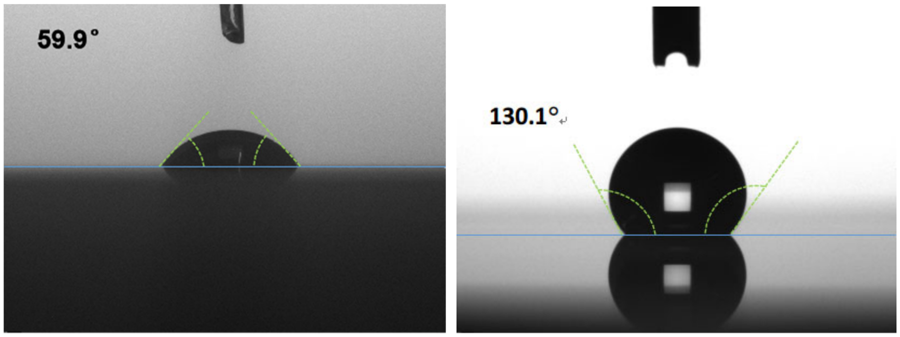

Figure S1. Water contact angle of K9 glass before and after coated with SiO_2 antireflective film.

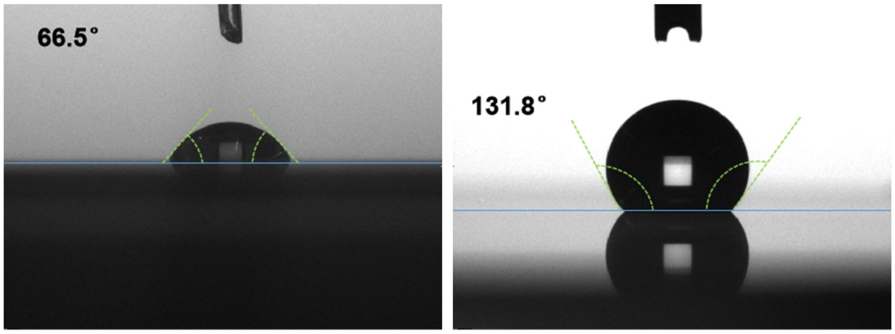

Figure S2. Water contact angle of Silicon substrate before and after coated with SiO_2 antireflective film.
